# Predictors of Insight in Pediatric OCD: The Impact of Autistic Traits, Age, and Symptom Dimensions

**DOI:** 10.1192/j.eurpsy.2026.10642

**Published:** 2026-07-31

**Authors:** R. Fuller, K. Prasanna, O. Consortium, M. Grados

**Affiliations:** 1 Johns Hopkins University, Baltimore, United States; 2 Jawaharlal Nehru Medical College, Belagavi, India; 3 Johns Hopkins University School of Medicine, Baltimore, United States

## Abstract

**Introduction:**

Obsessive-compulsive disorder (OCD) is characterized by intrusive, unwanted thoughts (obsessions) and rigid, ritualistic behaviors (compulsions). Insight in OCD refers to the recognition of the external nature of the symptoms through the identification of their irrational and unreasonable nature.

**Objectives:**

1) To test the association of autistic traits and insight in OCD using a pediatric OCD sample; 2) to assess the association of insight with OCD severity and OCD symptom dimensions.

**Methods:**

The sample was derived from the OCD Collaborative Genetics Association Study (OCGAS). Inclusion criteria were: 1) youth ages 8-17 years; 2) primary diagnosis of OCD; 3) proband in the family sample. Exclusion criteria were diagnoses of autism, Tourette syndrome, primary mood or psychotic disorder. Insight used a Likert scale (1-4): excellent, good, fair, poor/absent. Autistic traits used the parent-rated Social Responsiveness Scale (SRS). Anova and T-tests analyzed frequency data, and linear regression were used for continuous data. Outlier identification and exclusion used the Interquartile Range (IQR) Method. OCD severity used the Yale-Brown Obsessive Compulsive Scale (YBOCS) scores, and symptom dimensions were extracted as factors using a principal components factor analysis approach.

**Results:**

172 youth (ages 8–17, mean 13; 53% male) comprised the OCGAS subset sample. Younger age was associated with poorer insight (F=2.6, p=0.053), while higher SRS total scores (autistic traits) also predicted poorer insight (β=0.156, p=0.04, Figure 1).

When controlling for age, the strength of the relationship between autism traits and poorer insight increased (β=0.167, p=0.03), and it was stronger in adolescents (β=0.186, p=0.04) than school-aged participants (β=0.160, p=0.3). SRS Social Cognition (β=0.201, p=0.01), Communication (β=0.173, p=0.02) were significant but not Repetitive Phenomena (β=0.149, p=0.06). There was no association between CY-BOCS severity and insight in this sample.

Aggressive and sexual obsessions, as well as checking and counting compulsions, are associated with relatively better insight, while contamination obsessions have a trend with worse insight (p=0.06). When examining OCD dimensions, the Taboo factor (aggressive, sexual, religious, checking) predicted better insight (β=-0.143, p=0.06), while contamination-cleaning predicted worse insight (β=0.140, p=0.07). Hoarding and the symmetry-counting-ordering-miscellaneous factor are not associated with insight.

**Image 1:**
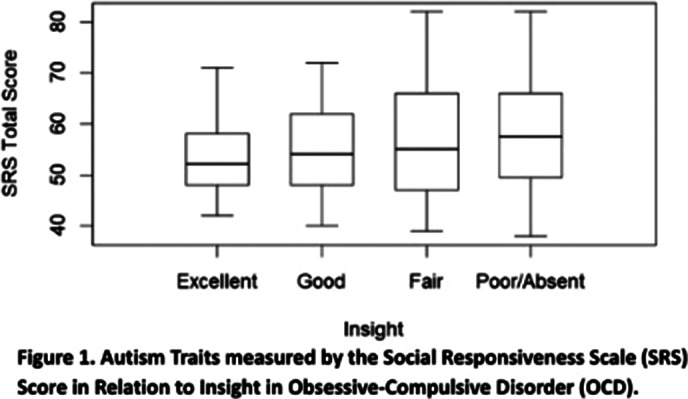
Image 1: Long description.

**Conclusions:**

Younger age and higher autistic traits predict worse insight in pediatric OCD; in particular, social cognition autistic traits seem to have the most impact. The capacity to self-reflect and discriminate on experiences such as obsessions or compulsions may be deficient in those with higher autistic characteristics, with theory of mind deficits possibly playing a role in the relative lack of self-reflection.

**Disclosure of Interest:**

None Declared

## Long descriptions

### Image 1: Long description

The Y-axis is labeled S R S Total Score with numerical increments of 10 from 40 to 80. The X-axis is labeled Insight and contains four categories from left to right: Excellent, Good, Fair, and Poor forward slash Absent.

Four box plots represent the data for each category:

* Excellent: The median is approximately 52. The box ranges from roughly 48 to 58, with whiskers extending from 42 to 71.

* Good: The median is approximately 54. The box ranges from roughly 48 to 62, with whiskers extending from 40 to 72.

* Fair: The median is approximately 55. The box ranges from roughly 47 to 66, with whiskers extending from 39 to 82.

* Poor forward slash Absent: The median is approximately 58. The box ranges from roughly 50 to 66, with whiskers extending from 38 to 82.

The trend shows a slight increase in the median S R S Total Score as Insight quality decreases from Excellent to Poor forward slash Absent.

Navigate back to Image 1:.

